# Mental Status after West Nile Virus Infection

**DOI:** 10.3201/eid1208.060097

**Published:** 2006-08

**Authors:** Kathleen Y. Haaland, Joseph Sadek, Steven Pergam, Leonor A. Echevarria, Larry E. Davis, Diane Goade, Joanne Harnar, Robert A. Nofchissey, C. Mack Sewel, Paul Ettestad

**Affiliations:** * New Mexico Veterans Affairs Healthcare System, Albuquerque, New Mexico, USA;; † University of New Mexico, Albuquerque, New Mexico, USA;; ‡ University of Washington, Seattle, Washington, USA;; § Northwest Mississippi Regional Medical Center, Clarksdale, Mississippi, USA;; ¶ New Mexico Department of Health, Santa Fe, New Mexico, USA

**Keywords:** West Nile virus, cognitive manifestations, cognitive symptoms, mental tests, dispatch

## Abstract

Mental status after acute West Nile virus infection has not been examined objectively. We compared Telephone Interview for Cognitive Status scores of 116 patients with West Nile fever or West Nile neuroinvasive disease. Mental status was poorer and cognitive complaints more frequent with West Nile neuroinvasive disease (p = 0.005).

West Nile virus (WNV) outbreaks have been studied in Africa since 1937 and in the United States since the initial New York City outbreak in 1999 (*1*). Studies of these outbreaks typically include only hospitalized patients, use retrospective medical chart reviews, and do not include follow-up after discharge (*1**–**5*). Therefore, the long-term sequelae of WNV are largely unknown.

Mental status after West Nile virus infection is an important public health issue because many studies of hospitalized patients have reported continued complaints from the time of discharge (*2**,**4**,**5*) through 18 months later (*6*). A limitation of these studies is their reliance on self-report of cognitive deficits rather than objective examination. No study of WNV patients has used objective assessment of mental status to determine the severity of cognitive deficits after acute WNV infection in a large sample of WNV patients, despite the fact that altered mental status is reported in 46% (*1**,**4*) to 74% (*3*) of WNV patients at the time of discharge from the hospital. In addition, no studies have determined whether mental status changes are more prevalent in patients who had West Nile neuroinvasive disease (WNND) than in patients who had West Nile fever (WNF), which would be expected, given the diagnostic criteria and the reports of less frequent and less severe cognitive deficits from WNF patients (*7*).

The purpose of our study was to objectively compare mental status of patients with a diagnosis of WNND or WNF, 9 months after symptom onset. We used the Telephone Interview for Cognitive Status (TICS) and subjective cognitive complaints noted during interview. Of the 190 eligible patients, all were seropositive for WNV and all had been reported to the New Mexico State Department of Health in 2003 (*8*). We successfully contacted 129 (68%) of these patients by telephone and excluded 13 who had received a diagnoses of a neurologic condition before the diagnosis of WNV infection or who did not speak English well. For the remaining 116 patients, diagnosis of WNND or WNF was made without knowledge of TICS score and was based on the reporting physician's diagnosis or medical record review for patients who were hospitalized for WNV infection or who had continuing neurologic or cognitive symptoms at the time of interview. Patients were evaluated with the TICS, which is highly correlated with the Mini Mental Status Examination (*9*), is sensitive to mental status deficits in the elderly (*10**,**11*), and is standardized for administration by telephone (*12*).

Table 1 shows that the WNF and WNND groups were comparable in age, sex, and ethnicity (p>0.05). However, because of a trend for lower education in the WNND group (p = 0.05), education was a covariate in all analyses. Analysis of covariance (ANCOVA) showed TICS total score to be poorer for the WNND than the WNF group (p = 0.005). Thus, a small, but consistent, effect suggests that WNV infection severity affects mental status.

**Table 1:** Demographic data from Telephone Interview for Cognitive Status*†

Characteristic	West Nile fever, n = 64	West Nile neuroinvasive disease, n = 52	p value
Age, y	50.0 (12.8)	53.6 (19.1)	0.26
Education, y	14.6 (2.6)	13.6 (3.0)	0.05
Sex, % male	44	58	0.14
Ethnicity			
% white	62	64	0.90
% Hispanic	33	31	0.90
Hospitalized, n (%)	10 (16)	40 (78)	<0.001
TICS total (range 0–41)	33.6 (3.3)	31.1 (3.8)	0.005‡

*TICS, Telephone Interview for Cognitive Status. †Means with standard deviations in parentheses, except where otherwise indicated. ‡p value, after analysis of covariance, controlling for marginal group difference in education.

Participants were also asked questions about current cognitive functioning (Table 2). Frequency of self-report of cognitive problems varied from 6% to 42% across both groups. Logistic regression, when controlled for education, showed reports of concentration difficulty (p = 0.05) and confusion (p = 0.02) to be significantly higher in the WNND group. Overall, the WNND group reported more cognitive problems than the WNF group (1.6 vs. 0.8, respectively, p = 0.009), and the number of cognitive problems was correlated with the TICS total score (r = -0.21, p = 0.02). These findings indicate that self-reported cognitive problems increased with severity of WNV infection.

**Table 2:** Percentage of patients reporting current cognitive problems

Mental deficit	West Nile fever	West Nile neuroinvasive disease	Overall
Concentration	22	42*	31
Memory	28	42	35
Understanding	11	27	18
Decision making	16	25	20
Confusion	6	25*	15
Mean rate of complaints	17	32	24

*p<0.05, after logistic regression, controlling for marginal group difference in education.

To determine the better predictor of WNV diagnostic category—TICS total score, rate of cognitive problems, or a combination—we performed a logistic regression predicting WNV diagnostic category from TICS total and rates of self-report of cognitive problems. Only the TICS total score significantly predicted WNND group membership (p = 0.01), but rate of report of cognitive problems was a marginal predictor (p = 0.07).

This is the first study to objectively measure mental status after WNV infection except for 1 review paper that mentioned a study that performed neuropsychological evaluation of WNV patients while they were hospitalized with acute infection (*13*). We show that 9 months after infection, WNND produces subtle but consistently greater mental status deficits than WNF. These findings are consistent with those of studies that identified a high incidence of cognitive problems from WNV patients and lesser complaints from WNF patients (*7*) from time of hospital discharge through 18 months later (*2**,**4**,**6**,**14*). We found subtle cognitive deficits in the WNND group that could not be explained by demographic variables. Although these cognitive differences are subtle, they suggest that WNND produces cognitive deficits after the acute symptoms have largely dissipated. Our data may underestimate the incidence of cognitive changes associated with WNND because more sensitive comprehensive neuropsychologic evaluations were not done.

Similar to previous studies (*5**,**6**,**14*) of chronic cognitive complaints after WNV infection, our study showed a high incidence of cognitive complaints, although subjective self-reports can be unreliable. Our data show that although ≈24% of the WNV patients complained of cognitive problems, complaints were somewhat greater for patients in the WNND group than in the WNF group. In addition, subjective reports of cognitive problems are only marginally associated with poorer mental status. This finding further supports the need to perform objective mental status examinations, especially because normal variation in cognitive performance can be misattributed to a medical diagnosis (*15*).

We were not able to determine whether the WNF group demonstrated cognitive deficits because we did not include a healthy control group and because TICS does not have normative data for respondents <60 years of age. However, the published norms for TICS recommend a cutoff score of >33 for classification as "normal" and <25 as clearly "impaired"; only 53% of our total sample fell into the normal range, despite being younger than the age for which norms are published (*9*). Furthermore, 33% of the WNF group scored in the abnormal range, suggesting that WNF may produce cognitive deficits relative to published norms. Although the influence of demographic differences (e.g., education) between the WNF and the normative group cannot be ruled out, the high incidence of abnormal scores in the WNF group may also reflect undiagnosed neuroinvasion of WNV. However, without a demographically matched control group, this question cannot be addressed definitively. In addition, 65% of the WNND group scored in the abnormal range, consistent with our other findings that WNND is associated with chronic mental status changes.

Our study has several advantages, including objective assessment of mental status, sampling from the entire state's reported cases of WNV infection in 1 year, direct comparison between WNF and WNND groups, and inclusion of patients of minority race and ethnicity. One potential limitation is the use of the reporting physician's diagnosis, but medical records were obtained for 78% of those at greatest risk for WNND. In all instances in which diagnosis was changed on the basis of medical records, WNF diagnosis was changed to WNND. Therefore, if we misclassified patients, we are more likely to have included in the WNF group patients who should have been in the WNND group; this potential bias would have decreased group differences by lowering the WNF mental status score.

These results emphasize that objective mental status assessment is more sensitive than subjective report and suggest that future studies should assess potential mental status deficits to clarify the long-term public health consequences of WNV.
